# Voice Cloning Using AI vs Traditional Audio Recording for Prerecorded Courses in Medical Pedagogy: Randomized Controlled Trial

**DOI:** 10.2196/86569

**Published:** 2026-07-02

**Authors:** Antoine Gavoille, Fabien Subtil, Mikaïl Nourredine

**Affiliations:** 1 Laboratoire de Biométrie et Biologie Evolutive Université Claude Bernard Lyon 1 Lyon, Rhône-Alpes France; 2 Service de Biostatistiques-Bioinformatiques Hospices Civils de Lyon Lyon France

**Keywords:** medical pedagogy, prerecorded course, generative artificial intelligence, voice cloning, audio quality, artificial intelligence, AI

## Abstract

**Background:**

Prerecorded courses are increasingly used in medical education, and audio quality is known to influence learners’ comprehension and engagement. Traditional audio recording, however, is time-consuming and may be uncomfortable for some educators. Advances in generative artificial intelligence (AI) now allow for realistic voice cloning, but its pedagogical value compared with conventional recording has not been assessed.

**Objective:**

This study aimed to evaluate the usefulness and perception of AI-based voice cloning for prerecorded courses in medical pedagogy compared with traditional audio recording.

**Methods:**

We conducted a randomized trial among fourth- and fifth-year medical students at a French university. Participants accessed four 10-minute prerecorded lectures on critical appraisal of medical research. The control group received lectures with audio recorded by the teacher, whereas the intervention group received audio generated from the teacher’s cloned voice using a commercial AI text-to-speech system, with identical slides and scripts. The primary outcome was the total score on 2 online tests (17 multiple-choice questions on knowledge acquisition and 15 multiple-choice questions on knowledge application). Secondary outcomes included satisfaction ratings, course viewing metrics, and production time.

**Results:**

A total of 88 students were randomized, and 64 (72.7%) watched at least 15 seconds of video and, thus, were included in the modified intention-to-treat population. Mean total test scores did not differ significantly between the AI voice cloning and audio recording groups (51.2, SD 4.9 vs 51.8, SD 6.9 out of 100; adjusted mean difference −0.9, 95% CI −2.7 to 4.4; *P*=.60). Satisfaction was high in both groups. Production time was shorter with AI (22.5, SD 6.45 vs 35, SD 7.07 minutes per video).

**Conclusions:**

We did not detect a significant difference in learning outcomes or satisfaction between AI voice cloning and conventional recording, whereas AI voice cloning reduced production time, making it a practical alternative for prerecorded medical courses. Nevertheless, some students may perceive synthetic voices as less authentic, representing a potential barrier to widespread adoption.

## Introduction

The growing integration of online resources into medical education has led to an increasing reliance on prerecorded video courses [1,2]. This evolution, accelerated by the COVID-19 pandemic, has necessitated a rapid transition to virtual learning environments. Consequently, it is essential to ensure the effectiveness and quality of online education to meet the needs of modern learners.

Audio quality is a key factor contributing to the success of online learning. Audio is the primary channel for conveying information in prerecorded courses, and audio quality can affect a student’s ability to understand the material and stay engaged. Furthermore, the perceived audio quality could influence a student’s unconscious assessment of the overall quality of a video [3]. Research on cognitive fluency, although not in an educational context, has shown that, when audio is difficult to process due to extraneous variables such as poor quality, it can lead to less favorable evaluations of the speaker and content and even influence the memorization of key facts [4,5]. This underscores the importance of considering audio quality as a critical variable in educational content.

The traditional method of recording audio for prerecorded courses presents several challenges for educators. It is a time-consuming process that requires access to studios and specialized equipment to achieve high quality. In practice, however, many teachers record on their PCs, using low-quality microphones and working in environments that are not always favorable to good sound capture. In addition, some educators may not be comfortable with the recording process, leading to diction or speech issues that can negatively impact the learning experience. Emerging technologies such as voice cloning using artificial intelligence (AI) offer a promising solution to these challenges [6]. This technology allows for the creation of a high-quality prerecorded audio track that retains the familiar voice of the educator, which students can recognize. With the recent advances in AI and deep learning, voice synthesis technology is evolving rapidly and can now produce voices so realistic that it is often impossible to distinguish them from actual human speech [7,8]. This approach has several advantages, including the ability to reduce production time and ensure consistent, high-quality sound without the need for a physical studio [9]. In addition, it allows for the correction of speech imperfections, improving clarity and intelligibility. However, no studies have specifically evaluated modern AI voice cloning for prerecorded lectures.

The aim of this study was to evaluate the usefulness and perception of AI voice cloning for generating audio in prerecorded video courses compared with traditional voice recording in medical training. The primary objective was to compare student learning outcomes between the 2 methods; secondary objectives included satisfaction, engagement, and production time. We hypothesized that traditional recording might perform better than AI-generated voice cloning in terms of student learning outcomes and satisfaction, whereas AI voice cloning would offer practical advantages, including reduced production time.

## Methods

### Study Design

This was a single-center randomized study conducted at a French university. Given the nature of the study, full blinding was not feasible, so students were aware that they were participating in a study but were not informed that the purpose of the study concerned the quality of the audio recordings to preserve ecological validity and avoid attention bias. Neither the participants nor the researchers responsible for analyzing the data were aware of the group assignments. All prerecorded lectures and assessments were hosted in a single online environment, the Moodle platform, an institutional learning management system used by the University of Lyon. Moodle provided a secure environment for delivering course materials, tracking user activity (total watch time and number of views), and collecting survey responses. Data were exported in anonymized form for analysis.

### Participants

Fourth- and fifth-year medical students were recruited on a voluntary basis to participate in this study. Volunteer students were randomized into 2 groups at a 1:1 ratio at the start of the study stratified by year. Participants who did not have a stable internet connection could have one provided by the faculty if needed. The conditions under which participants viewed the prerecorded courses were not standardized, each student being free to watch the courses in their own uncontrolled environment.

### Ethical Considerations

All participating students provided written informed consent prior to inclusion and were informed that they could withdraw their consent at any time without consequence. At the end of the study, the objective was disclosed to all participants during a group debriefing session conducted via videoconference, and an in-person debriefing was offered but was not requested by any participant. Voice cloning was performed exclusively by the teacher (AG) using a personal ElevenLabs account under his full informed consent. ElevenLabs requires voice identity verification prior to cloning, ensuring that only the voice owner can initiate the process. The voice recordings used for cloning were processed by ElevenLabs as a data processor under its Data Processing Addendum in compliance with the European Union General Data Protection Regulation. According to ElevenLabs’ privacy policy, raw voice data are retained for up to 3 years after the last interaction; the teacher retained the right to request data deletion at any time. Beyond ElevenLabs as the processor, no other party had access to the voice data. The study protocol was approved by an ethics committee (*Comité d’Ethique de la Recherche* of the University of Lyon; reference: 2025 06-12-002). This report follows the CONSORT (Consolidated Standards of Reporting Trials) guidelines.

### Intervention

Participants had online access to 4 prerecorded video courses, each lasting approximately 10 minutes, on the topic of critical appraisal skills in medical research and epidemiology. They could watch the courses at their convenience as many times as they wanted during a period of 2 months. The slides and text scripts were identical for both groups, with the only difference being the nature of the audio. The control group received lectures with traditional audio recordings produced under optimized nonprofessional conditions representative of typical academic settings: a lapel RODE Lavalier GO microphone was used, the instructor followed basic recording guidelines, and nonexpert postproduction editing (noise reduction and level normalization) was applied without access to a professional recording studio. For the intervention group, the teacher’s voice was cloned using the Professional Voice Cloning system from ElevenLabs [10] trained on 30 minutes of audio recorded using the same microphone that was distinct from the course content itself. Audio was then generated by an AI text-to-speech tool, the Turbo 2.5 model from ElevenLabs [11], using exactly the same text script. The following generation parameters were applied consistently across all AI-generated audio files: speech rate of 1.15, similarity of 80%, and stability of 40%. Audio samples generated using the 2 methods are provided in Multimedia Appendix 1.

### Outcome

One month after access to the videos was granted, students were given access to 2 online tests: a knowledge acquisition test with 17 multiple-choice questions on the content of the prerecorded lectures and a knowledge application test with 15 multiple-choice questions on a medical research article. Each question included 5 items, with 1 point per correct answer. In addition, participants were asked to complete a satisfaction questionnaire immediately after viewing the course, evaluating overall satisfaction, audio quality, voice, and attention maintenance throughout the videos on a 4-point Likert scale. The time required for the teacher to produce the audio track and participants’ viewing metrics (duration and number of views) were also collected for each video.

The primary outcome was the total score on the 2 tests, expressed as a score out of 100. Secondary outcomes were the score on the knowledge acquisition and application tests separately, each item of the satisfaction questionnaire (averaged across all the prerecorded course videos watched by the participant), and the number of prerecorded course videos watched entirely (>90%). Finally, we evaluated the mean time required to produce the audio recording for each video, defined as the total active time from slides without audio to final video with recorded sound. For the traditional recording method, this included script drafting, audio recording (including failed takes), and postproduction editing. For the AI voice cloning method, this included refining the script into a precise written text (with particular attention to punctuation, which influences text-to-speech prosody) and audio-video synchronization; the audio generation step (approximately 2-3 minutes per video, requiring no instructor involvement) was excluded.

### Statistical Analysis

A power calculation was performed to determine the sample size required to detect a significant difference between the 2 groups. The calculation was based on a 2-sided hypothesis test with an α level of 5% and a power of 80%. For an expected score of 60 points in the control group with an SD of 15 points (based on an expected distribution of scores with a first quartile at 50 points and a third quartile at 70 points) to detect a minimum difference of 10 points between the control group and the intervention group and including a 20% loss to follow-up, a minimum of 45 participants per group was required for the study, for a total of 90 students.

Quantitative variables are presented as means and SDs, and categorical variables are presented as counts and percentages. Outcomes were analyzed in the modified intention-to-treat (mITT) population, corresponding to participants who watched at least 15 seconds of 1 prerecorded course video. This threshold was chosen based on viewing analytics showing a first sharp drop before this point, reflecting accidental or exploratory accesses without genuine viewing intent, and was also considered too short for a participant to consciously identify an AI-generated voice and disengage. For participants who responded to only 1 of the 2 knowledge tests (n=8), their total score was calculated from the available test alone, rescaled to 100 points using the same formula as for each individual test. This approach is equivalent to imputing the missing test score by the participant’s score on the completed test and assumes that performance on the missing test would have been, on average, equivalent to that on the completed test. As a sensitivity analysis, the primary analysis was repeated in the subgroup of participants who completed both tests (n=42). Other missing data were not imputed, and only complete cases were analyzed for each outcome. Knowledge test scores were analyzed using a linear regression adjusted for the intervention or control group and year of study. Other categorical outcomes were compared between the intervention and control groups using a nonparametric Fisher exact test. *P* values below .05 were considered statistically significant. Analyses were performed using the R software (version 4.4.2; R Foundation for Statistical Computing) [12].

## Results

### Overview

**Figure 1:**
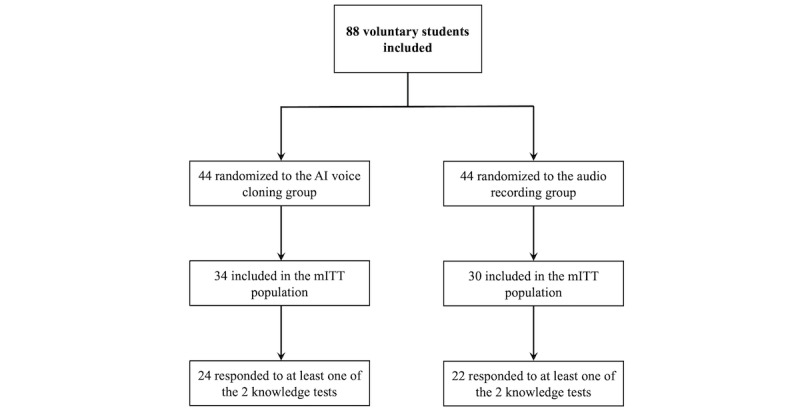
Study flowchart. The modified intention-to-treat (mITT) population was defined as the population who watched at least 15 seconds of a prerecorded course video. AI: artificial intelligence.

A total of 88 participants took part in June 2025, of whom 44 (50%) were randomized to the audio recording group and 44 (50%) were randomized to the AI voice cloning group; the study flowchart is shown in Figure 1. Of these 88 participants, 64 (72.7%) watched at least 15 seconds of 1 prerecorded course video and, thus, were included in the mITT population: 34 (53.1%) participants in the AI voice cloning group and 30 (46.9%) in the audio recording group. Participant characteristics by study group are shown in Table 1. In total, 71.9% (46/64) of the participants were women, and 68.8% (44/64) were in fifth year. Year of study was well balanced in the initially randomized population, whereas there were more fourth-year students in the AI voice cloning group in the mITT population. Regarding missing data, 78.1% (50/64) of the participants responded to at least one of the knowledge tests, including 12.5% (8/64) who responded to only 1 of the 2 tests, based on which their total score was calculated, and 93.8% (60/64) who responded to at least one satisfaction questionnaire.

**Table 1:** Participant characteristics and outcomes in the modified intention-to-treat population (N=64).

			Total	Randomization group	
				AI^a^ voice cloning (n=34)	Audio recording (n=30)
**Participant characteristics, n (%)**					
	Men		18 (28.1)	9 (26.5)	9 (30)
	**Year of study**				
		Fourth	20 (31.3)	7 (20.6)	13 (43.3)
		Fifth	44 (68.8)	27 (79.4)	17 (56.7)
**Primary outcome**					
	**Total score (out of 100)^b^**				
		Mean (SD)	51.5 (6.0)	51.2 (4.9)	51.8 (6.9)
		Median (IQR)	51.3 (48.1-55.0)	51.3 (48.1-53.8)	51.3 (48.8-56.0)
		Missing, n (%)	14 (21.9)	9 (26.5)	5 (16.7)
**Secondary outcomes**					
	**Knowledge acquisition test score (out of 100)**				
		Mean (SD)	53.5 (5.6)	54.3 (4.9)	52.6 (6.3)
		Median (IQR)	54.1 (49.4-56.5)	54.7 (50.6-58.8)	51.8 (48.2-56.5)
		Missing, n (%)	18 (28.1)	10 (29.4)	8 (26.7)
	**Knowledge application test score (out of 100)**				
		Mean (SD)	49.3 (8.7)	47.0 (6.0)	51.5 (10.2)
		Median (IQR)	47.3 (44.0-54.7)	46.7 (42.7-50.7)	50.0 (44.7-57.3)
		Missing, n (%)	18 (28.1)	12 (35.3)	6 (20)
	**Number of prerecorded course videos watched (>90%), n (%)**				
		0	2 (3.1)	1 (2.9)	1 (3.3)
		1	3 (4.7)	2 (5.9)	1 (3.3)
		2	5 (7.8)	2 (5.9)	3 (10)
		3	5 (7.8)	3 (8.8)	2 (6.7)
		4	49 (76.6)	26 (76.5)	23 (76.7)

^a^AI: artificial intelligence.

^b^Primary outcome: total score (out of 100) on the 2 knowledge tests (knowledge acquisition+knowledge application).

### Participants’ Results on the Knowledge Test Scores

Primary and secondary outcome results are shown in Table 1. The mean total score on the 2 knowledge tests was 51.2 (SD 4.9) points in the AI voice cloning group and 51.8 (SD 6.9) in the audio recording group, with no significant difference between the 2 groups (adjusted mean difference −0.9, 95% CI −2.7 to 4.4; *P*=.60). On the knowledge acquisition test, the mean score was 54.3 (SD 4.9) in the AI voice cloning group and 52.6 (SD 6.3) in the audio recording group (adjusted mean difference 1.2, 95% CI −2.2 to 4.6; *P*=.50). On the knowledge application test, the mean score was 47.0 (SD 6.0) in the AI voice cloning group and 51.5 (SD 10.2) in the audio recording group (adjusted mean difference −4.9, 95% CI −7.7 to 0.4; *P*=.07).

### Satisfaction Questionnaire

**Figure 2:**
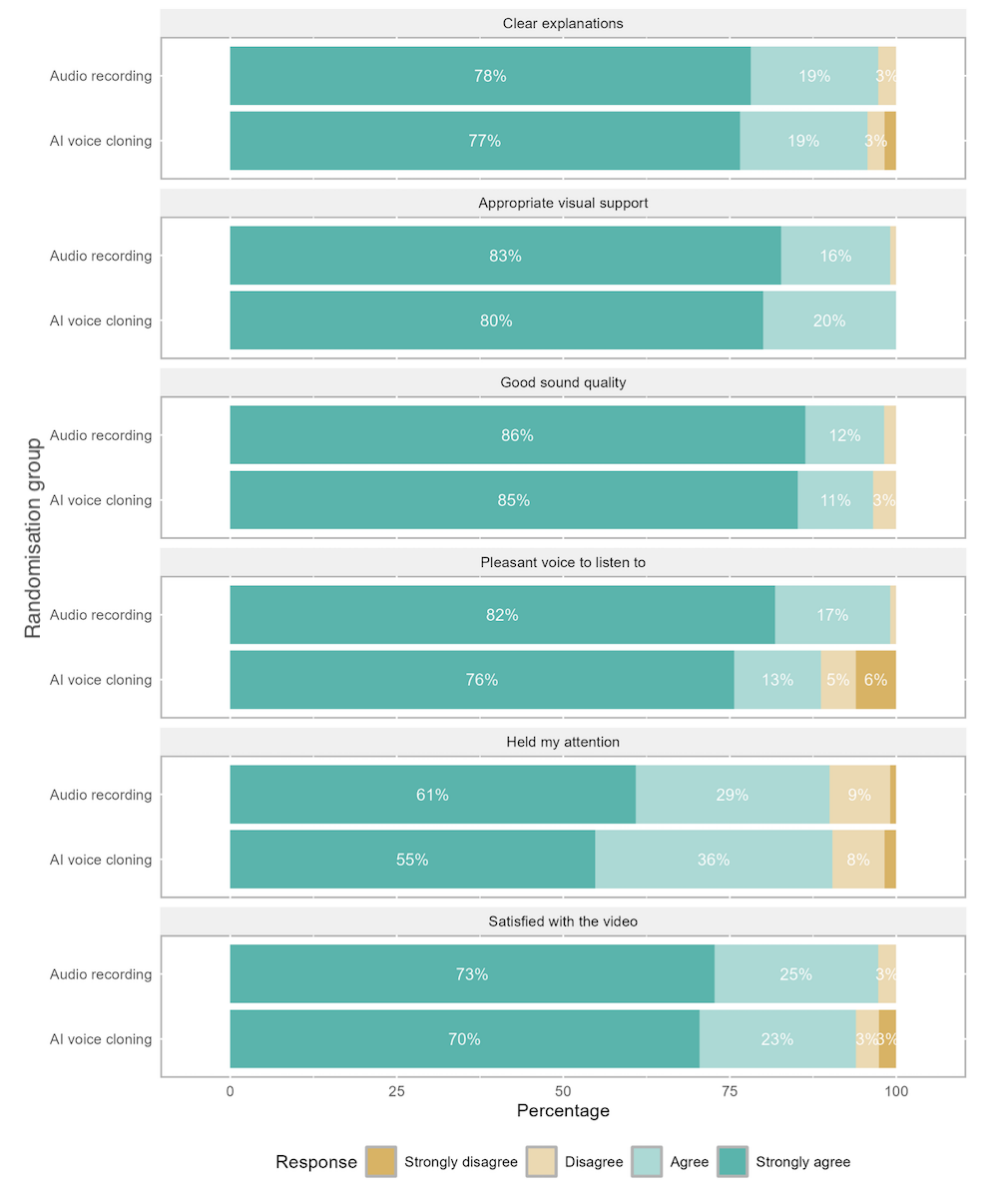
Satisfaction level by study group. AI: artificial intelligence.

Over the 4 prerecorded course videos, participants had an overall high satisfaction level. Averaged across participants, a mean proportion of 98% agreed or strongly agreed that the prerecorded course videos had good sound quality, 96% agreed or strongly agreed that the explanations were clear, 94% agreed or strongly agreed that the voice was pleasant to listen to, 90% agreed or strongly agreed that the video held their attention, and 96% agreed or strongly agreed that they were globally satisfied with the videos. We found no significant difference in the proportion of “agree” or “strongly agree” responses to satisfaction items between the AI voice cloning group and the audio recording group (Figure 2). However, a mean proportion of 11% disagreed or strongly disagreed that the voice was pleasant to listen to in the AI voice cloning group compared to 1% in the audio recording group, but the difference was not significant (*P*=.24). Satisfaction results for each prerecorded course video were similar to the overall satisfaction results (Multimedia Appendix 2).

### Other Outcomes

Overall, 76.6% (49/64) of the participants watched the 4 prerecorded course videos entirely, with no significant difference between the AI voice cloning and audio recording groups (*P*=.98). The AI voice cloning method was faster for generating the audio, with a mean of 22.5 (SD 6.45) minutes per video for AI voice cloning and 35 (SD 7.07) minutes per video for audio recording, representing a 36% reduction.

### Sensitivity Analysis

The results of the analysis restricted to participants who completed both tests (42/64, 65.6%) were consistent with those of the main analysis, with an adjusted mean difference of 2.3 in the total score between groups (95% CI −1.3 to 5.9; *P*=.20).

## Discussion

In this randomized controlled study comparing AI-based voice cloning with traditional audio recording for prerecorded medical courses, we found no significant difference in students’ knowledge acquisition and application, satisfaction, or engagement, whereas AI voice cloning allowed for a reduction in production time.

Recent advances in generative AI for text to speech have brought synthetic voices to a level of realism that can effectively compete with traditional human recordings. Modern systems are now capable of reproducing natural prosody and timbre with high fidelity, making it increasingly difficult for listeners to distinguish between cloned and real voices [8,13]. In this context, our results suggest that the substitution of human recordings through AI voice cloning did not compromise learning effectiveness. This stands in contrast to studies on degraded audio, which have shown that poor sound quality can impair comprehension, reduce trust in the speaker, and negatively affect performance [3,4]. In our study, both methods provided sufficiently clear audio to avoid such detrimental effects, indicating that the shift from traditional audio recording to AI synthesis did not perturb the educational process.

Student satisfaction was generally high across groups, confirming that audio quality, whether cloned or recorded, was adequate. It should be noted that traditional audio recordings were produced under carefully optimized real-world conditions, including the use of a lapel microphone and basic postproduction editing. The relative advantages of AI voice cloning are likely greater in settings with poorer recording conditions, such as built-in microphone use or no postproduction. Our results may therefore underestimate the practical benefit of voice cloning for educators with limited access to recording equipment or technical support. However, a nonsignificant trend suggested that a slightly lower proportion of students in the AI group found the voice pleasant. This aligns with prior observations that, despite advances in voice synthesis, subtle elements of natural prosody and emotional tone remain difficult to replicate [5]. Student perceptions of AI technology may also influence their satisfaction and, consequently, their attention and engagement if the “illusion” of naturalness is not fully achieved. In our study, while overall satisfaction remained high, one participant explicitly identified the voice as AI generated and expressed marked discontent. This reaction illustrates that, even when objective measures of learning are unaffected, individual opinions on AI can shape the learner’s experience. Such perceptions may represent an obstacle to the broader acceptance of voice cloning technologies in medical education, especially if students feel that artificiality compromises authenticity [14].

From a practical perspective, the reduced production time associated with AI voice cloning could represent a meaningful advantage for educators. Traditional recording often requires repeated takes, technical adjustments, and postprocessing to obtain an acceptable result, which can be discouraging for teachers who are not comfortable with oral performance or technical aspects of recording. Diction problems, hesitations, or lack of confidence can further extend the process and reduce the overall quality of the material. In our study, the time required to produce a 10-minute video was reduced by approximately one-third using AI synthesis. Beyond raw recording time, preparation and rerecording can accumulate into several hours for a series of courses, whereas AI allows for rapid corrections and consistent results with minimal additional effort.

AI voice cloning can also have additional advantages for educators. It could allow for switching of the cloned voice if a teacher is no longer involved in the course, thereby maintaining a sense of continuity and social connection between students and their current instructors. Moreover, these systems could integrate flexible options, for instance, adjusting the pace of speech while preserving natural prosody for students who wish to watch the course at a different speed rather than simply speeding up or slowing down the audio, which results in a deterioration of quality [15]. Finally, AI voice cloning could also enhance accessibility by providing clearer diction for students with hearing difficulties, tailored pronunciation for nonnative speakers, or even multilingual courses in languages that the lecturer does not speak [16], thus widening the inclusiveness of digital pedagogy. However, a limitation remains with respect to video: while audio can now be convincingly synthesized, generative technologies are not yet mature enough to produce realistic video sequences of the teacher speaking, although such developments are likely forthcoming [14,17].

Further research should explore how AI voice cloning interacts with other aspects of digital pedagogy, such as visual presence, interactivity, and feedback dynamics [15]. Experimental designs combining AI-generated audio with avatars or virtual instructors could help understand how different modalities influence motivation, empathy, and cognitive load [14,17]. Longitudinal studies are also needed to assess whether repeated exposure to synthetic voices affects learners’ engagement or recall. Finally, cost-effectiveness analyses could quantify the institutional benefits of AI-assisted production and inform decisions about large-scale adoption in medical schools.

The use of AI voice cloning in educational settings raises important ethical considerations regarding teachers’ consent and responsibility. In our study, voice cloning was performed by the teacher himself, who retained full control over the process and the resulting synthetic voice. We believe that this model, where the teacher whose voice is cloned is also the one operating the tool, represents the most ethical approach as it preserves both consent and accountability. Delegating voice cloning to a third party would raise substantially more complex questions regarding ownership, storage, and the potential for misuse of a person’s vocal identity. We therefore recommend that AI voice cloning in medical education be performed exclusively by the instructor themselves, who bears responsibility for both the content and the audio identity of the course material.

This study has several limitations. First, although the sample size calculation incorporated a 20% loss to follow-up margin, an additional 21.9% (14/64) of the mITT participants had missing data for the primary outcome, resulting in the analysis of only 78.1% (50/64) of the participants. This reduced the statistical power to detect small differences; however, the observed between-group difference was less than 1 point, well below the minimum clinically relevant threshold of 10 points, suggesting that the null result is not solely attributable to insufficient power. Furthermore, 12.5% (8/64) of the participants completed only 1 of the 2 knowledge tests; their total score was derived from the available test alone. Although the assumption of interchangeability between the 2 tests is supported by their conceptual proximity, this imputation remains imperfect given that the tests assess distinct constructs (knowledge recall vs knowledge application) and that total scores derived from a single test have higher measurement variance, which may introduce minor heterogeneity in the primary outcome measure. No data were collected on the reasons for nonviewing or early dropout, but this likely reflects the real-world study conditions: video viewing was voluntary and took place during a period without formal classes, when students were engaged in hospital internships and, for fifth-year students, intensive examination preparation. Although the complete-case analysis would yield an unbiased estimate under a missing completely at random or missing at random mechanism conditional on baseline covariates, a missing not at random mechanism related to group allocation cannot be excluded, for instance, if students who disengaged from the AI voice cloning group due to perceived artificiality were also less likely to complete the tests. A sensitivity analysis restricted to participants who completed both tests yielded consistent results. Second, although participants were not informed that the study concerned audio quality, blinding cannot be guaranteed as one participant explicitly identified the voice as AI generated. The study protocol included a poststudy questionnaire assessing whether participants had guessed their group allocation; however, the response rate was insufficient (6/64, 9.4%), and these data could not be reported. This is unlikely to have affected knowledge test scores, which were automatically graded, but may have influenced self-reported satisfaction outcomes. Third, the study was conducted in an uncontrolled environment, reflecting real-world conditions but introducing variability in viewing practices. Fourth, no baseline knowledge assessment was performed before the intervention, which would have improved statistical precision. This design choice was made deliberately to limit participant burden in the context described above. Finally, our study was designed as a superiority trial, so the absence of a statistically significant difference does not demonstrate equivalence between methods. The 95% CI excluded the prespecified minimum clinically relevant difference, suggesting that AI voice cloning may represent a practical alternative for prerecorded medical courses. However, the knowledge application test showed a nonsignificant trend favoring traditional audio, which could suggest that subtle differences in prosody or intonation might influence higher-order reasoning; this warrants further investigation. Future studies should explore the impact of voice cloning in larger, more diverse populations and in different pedagogical contexts.

In conclusion, our trial did not detect a significant difference in learning outcomes or satisfaction between AI-based voice cloning and traditional recording for prerecorded course videos in medical pedagogy. While AI-based voice cloning does not improve learning outcomes compared with human audio, it offers a practical advantage in terms of efficiency without compromising student satisfaction or knowledge acquisition. However, its adoption may still face resistance as some students remain sensitive to the artificial nature of synthetic voices and may perceive them as less authentic. Addressing this challenge of global acceptance will be essential to integrate AI-generated audio into medical education.

## Appendix

Multimedia Appendix 1 Samples of audio recording and artificial intelligence (AI) voice cloning. Short video presenting 2 consecutive audios from one of the prerecorded lectures: the traditional recording (0:01-0:07) followed by the AI voice cloning version (0:07-0:13).

Multimedia Appendix 2 Satisfaction level by study group for each prerecorded course video.

Multimedia Appendix 3 CONSORT 2025 checklist.

## Abbreviations

AI: artificial intelligence
CONSORT: Consolidated Standards of Reporting Trials
mITT: modified intention-to-treat

## Data Availability

Data are available from the corresponding author on reasonable request.
