# Supplementary material for: Voice Cloning Using AI vs Traditional Audio Recording for Prerecorded Courses in Medical Pedagogy: Randomized Controlled Trial
Source: JMIR Med Educ. 2026 Jul 2;12:e86569. doi: 10.2196/86569 (PMC13376843; doi:10.2196/86569)

**Multimedia Appendix 2. Satisfaction level according to study group for each pre-recorded course**


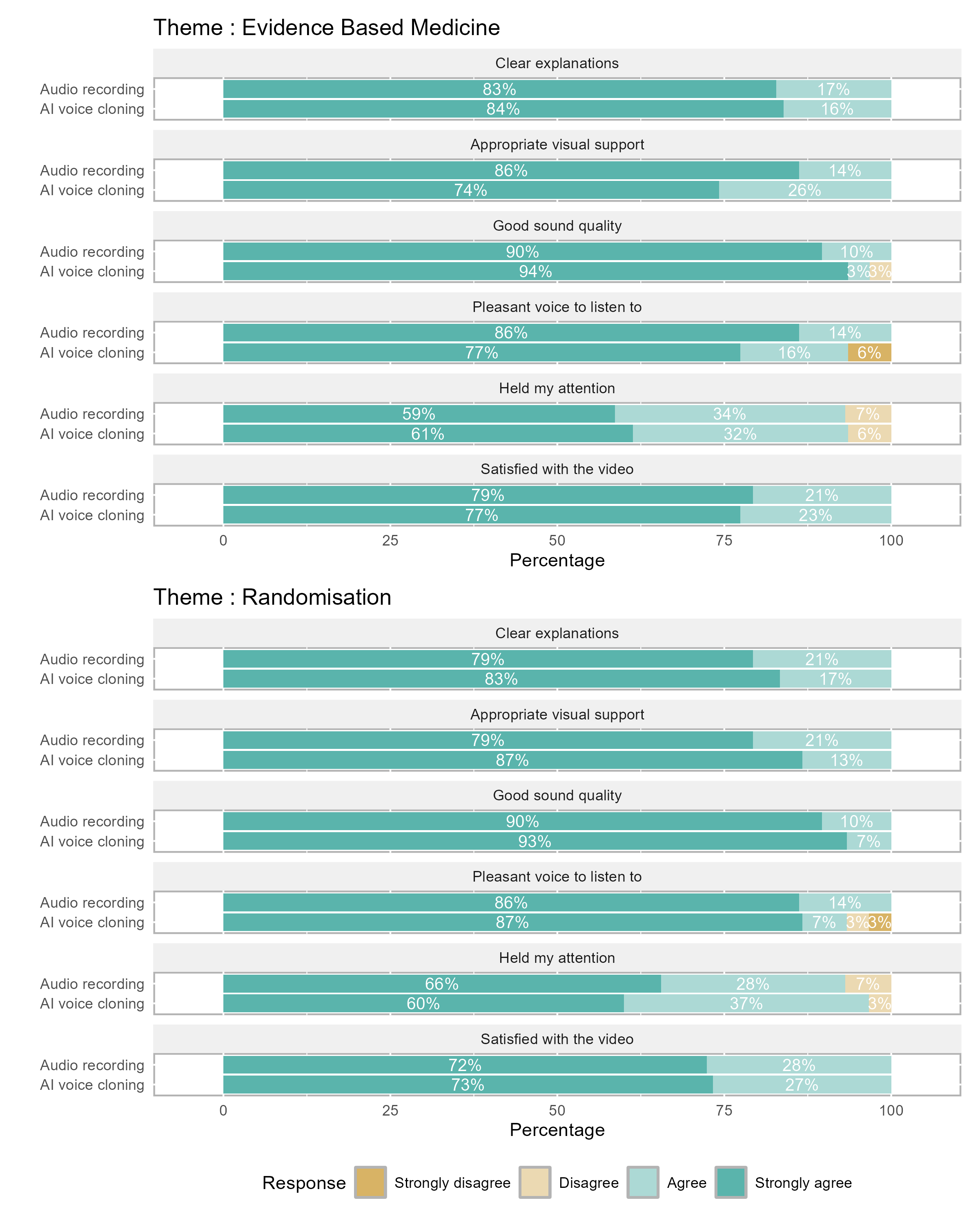

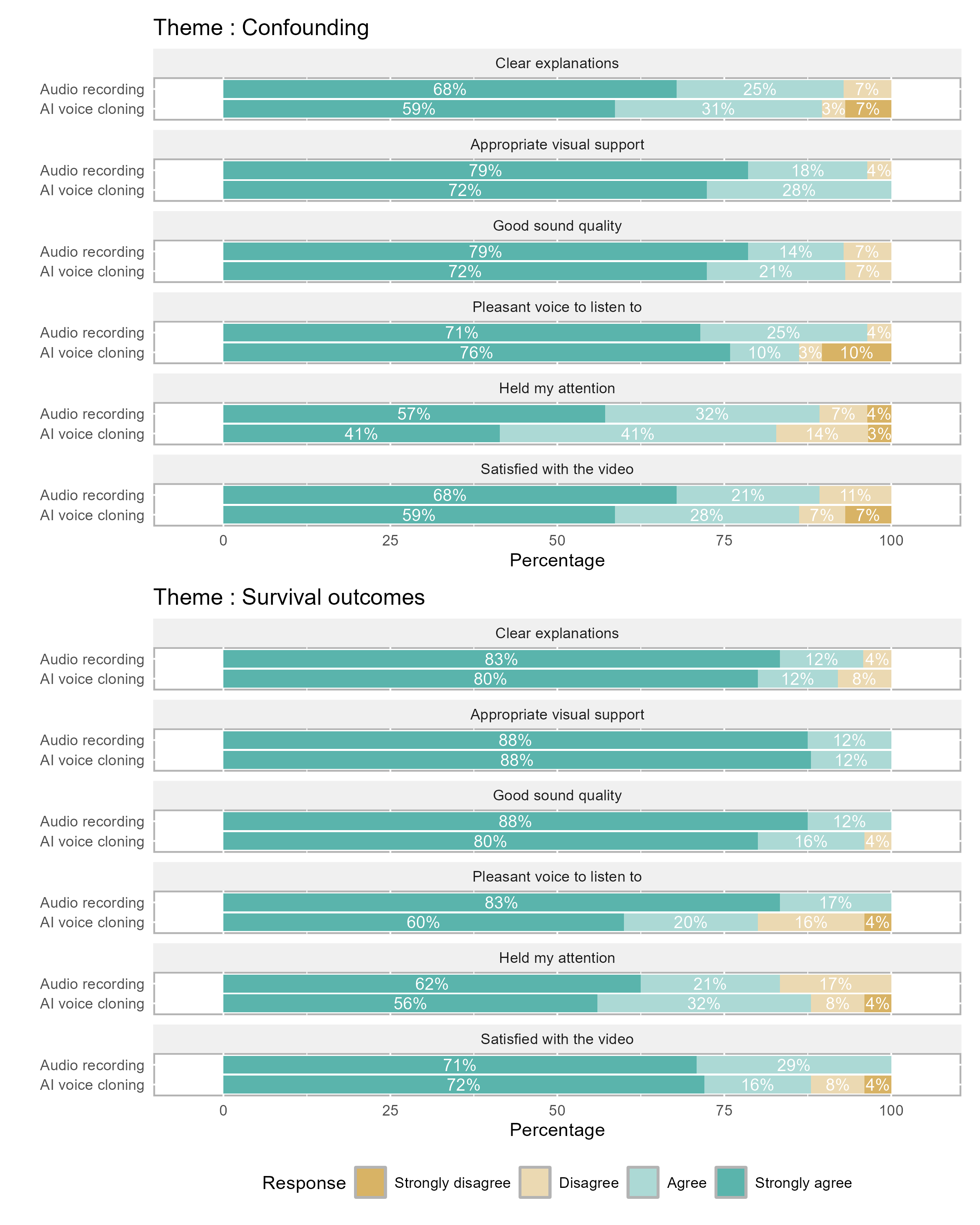

Supplement: Multimedia Appendix 2 [file mededu_v12i1e86569_app2.docx]
